# Isolated pancreatectomy using mesenteric approach

**DOI:** 10.1002/jhbp.1092

**Published:** 2021-12-21

**Authors:** Akimasa Nakao

**Affiliations:** ^1^ Department of Surgery Nagoya Central Hospital Nagoya Japan

**Keywords:** catheter‐bypass procedure of the portal vein, isolated pancreatoduodenectomy, mesenteric approach, non‐touch isolation, pancreatic cancer

## Abstract

In 1981, we developed the first antithrombogenic bypass catheter for the portal system. This catheter‐bypass procedure relieved the time limitation caused by portal occlusion and facilitated safe and easy resection and reconstruction of the portal vein or hepatic artery. We thereafter explored isolated pancreatoduodenectomy, in which pancreatoduodenectomy is performed under non‐touch isolation techniques. It is difficult to perform isolated pancreatoduodenectomy because of the complex arterial anatomy of the peripancreatic head region. In 1992, a mesenteric approach was developed for pancreatoduodenectomy. This approach allows dissection from the non‐cancerous side and determination of both cancer‐free margins and resectability followed by systematic lymphadenectomy around the superior mesenteric artery. This approach also enables early ligation of the inferior pancreatoduodenal artery and dorsal pancreatic artery branches from the superior mesenteric artery, as well as complete excision of the total mesopancreas (which is thought to be the second portion of the pancreatic head nerve plexus). Through this development of the mesenteric approach and antithrombogenic catheter‐bypass procedure, our isolated pancreatoduodenectomy was finally established in 1992. This is the ideal surgery for pancreatic head cancer from both surgical and oncological aspects. We herein introduce the precise surgical techniques.

## Graphical abstract

**Highlight**

Nakao established isolated pancreatoduodenectomy for pancreatic head cancer through the development of the antithrombogenic catheter‐bypass procedure of the portal vein and mesenteric approach in 1981. The history and precise surgical techniques of these procedures, and the surgical and oncological merits of this operation are introduced by the originator Nakao himself.

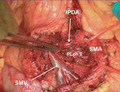

## 1 INTRODUCTION

Pancreatoduodenectomy (PD) for pancreatic head cancer under non‐touch isolation techniques has been a difficult procedure because of the complex vascular anatomy of the pancreatic head region. In 1981, we developed a breakthrough antithrombogenic bypass catheter for the portal vein (PV) system to prevent portal congestion or hepatic ischemia during PV resection and facilitate concurrent resection of the hepatic artery.^1^, ^2^, ^3^, ^4^, ^5^, ^6^, ^7^ This technique was performed by bypassing the congested portal blood from one branch of the superior mesenteric vein (SMV) into the femoral vein during PV resection and reconstruction, thus preventing portal congestion. Additionally, the blood flow into the intrahepatic PV through the umbilical vein in the hepatic round ligament could prevent both portal congestion and hepatic ischemia during concurrent resection and reconstruction of the PV and hepatic artery. This ideal procedure relieved the time limitation during surgeries involving portal occlusion. Since then, we have been aggressively resecting advanced pancreatic cancer with portal invasion using this catheter‐bypass procedure.^8^, ^9^, ^10^, ^11^ This antithrombogenic catheter is now commercially available, and the technique has become widespread throughout Japan. Along with our mesenteric approach, this procedure is one of the essential techniques when the isolated PD is performed in patients with locally advanced pancreatic cancer.

After developing this catheter‐bypass procedure, we attempted to aggressively resect pancreatic cancer with PV invasion using Kocher's maneuver^12^ in the 1980s. That is, the first step in PD was typically Kocher's maneuver. However, we sometimes encountered pancreatic cancer complicated by PV obstruction with well‐developed collateral veins in these surgeries using Kocher's maneuver, and we unfortunately struggled with massive bleeding despite the fact that we applied the catheter‐bypass procedure for the congested PV. At this time, we became acutely aware that Kocher's maneuver was not applicable to such situations. Therefore, we began to explore the use of isolated PD for pancreatic head cancer.

In cancer surgery, “isolated” refers to en bloc resection using a non‐touch isolation technique. In our isolated PD, all arteries that supply the pancreatic head region are first ligated and divided, and all drainage veins in this region are then ligated and divided before manipulation of the pancreatic head cancer. We actually established this procedure after development of the catheter‐bypass procedure and found that the first step in PD should be clearance of the mesenteric root instead of Kocher's maneuver. Our first step in PD is application of the mesenteric approach; that is, we never perform Kocher's maneuver. The mesenteric approach involves clearance of the connective tissues around the SMV and superior mesenteric artery (SMA) in the mesenteric root, which means systematic lymphadenectomy is performed around the SMA. We start at the mesenteric incision, extending from the ligament of Treitz to the lower border of the second portion of the duodenum. All branches from the SMA root, including the middle colic artery (MCA), second jejunal artery (JA2), first jejunal artery (JA1), inferior pancreatoduodenal artery (IPDA), dorsal pancreatic artery (DPA), and replaced right hepatic artery (rRHA), are gradually exposed during this approach. In this procedure, all arterial branches from the SMA supplying the pancreatic head region are totally ligated and divided. This approach also allows for total excision of the mesopancreas^13^; in other words, the second portion of the pancreatic head nerve plexus (PLphII)^14^, ^15^ (i.e. the so‐called “SMA margin”) is completely excised. Thus, our isolated PD procedure was finally established as described above, and we published this original operative method in Japanese in 1992^16^ and in English in 1993.^17^

We believe that this isolated PD procedure is the most important technique to obtain a cancer‐free surgical margin in PD for pancreatic head cancer. Furthermore, the use of the mesenteric approach makes it easy to reconstruct the PV by end‐to‐end anastomosis. In this way, both the development of the catheter‐bypass procedure and use of the mesenteric approach have made it possible to perform isolated PD with PV resection easily and safely.^18^, ^19^, ^20^, ^21^, ^22^, ^23^, ^24^, ^25^

## 2 ANTITHROMBOGENIC CATHETER‐BYPASS PROCEDURE OF THE PV

**FIGURE 1:**
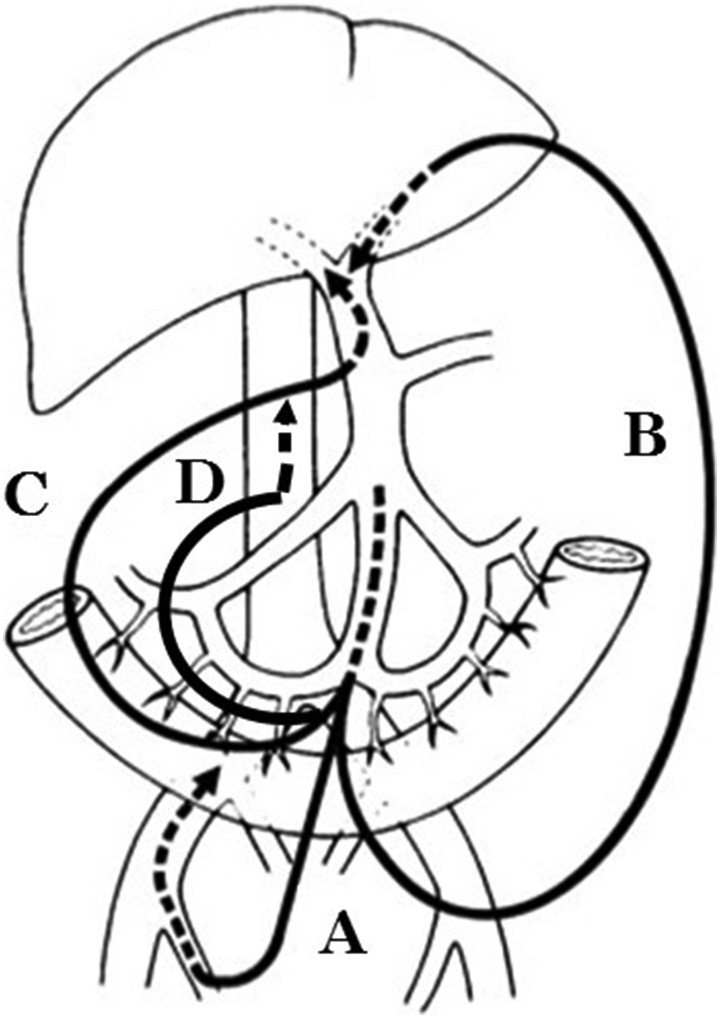
Catheter‐bypass procedure of the portal vein (from Reference^6^). A, Bypass between the mesenteric and femoral veins. B, Bypass between the mesenteric and umbilical veins. C, Bypass between the mesenteric and hepatic hilar portal veins. D, Bypass between the mesenteric vein and inferior vena cava

**FIGURE 2:**
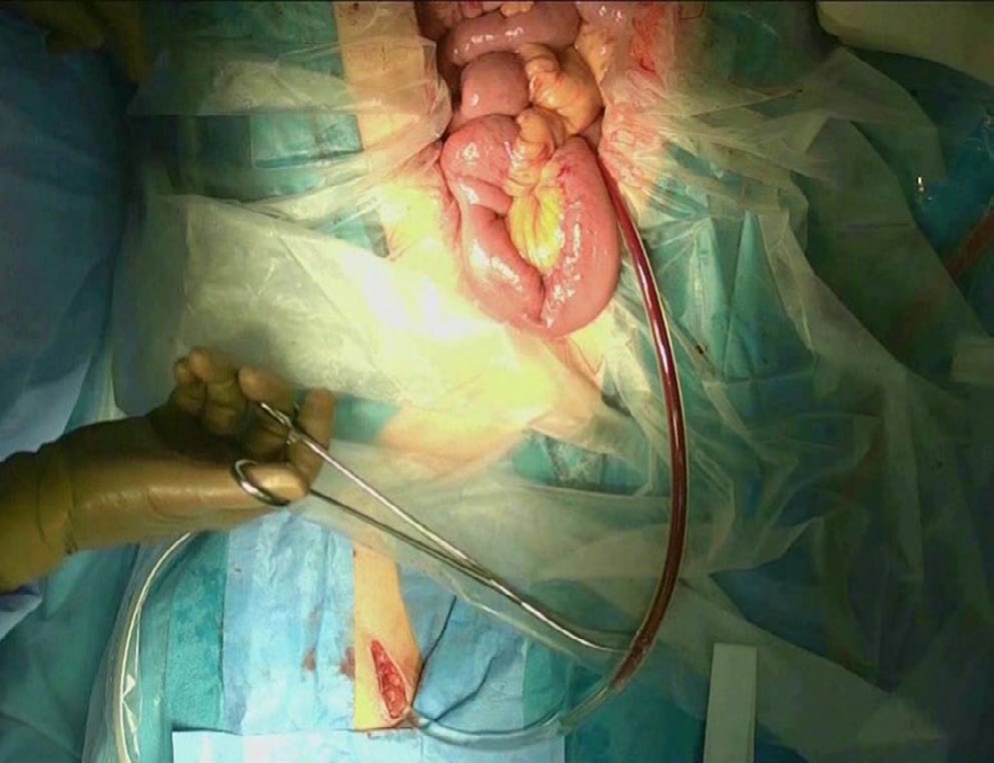
Catheter‐bypass procedure of the portal vein between a branch of the superior mesenteric vein and right femoral vein

In the 1970s, PV resection during PD for pancreatic head cancer was thought to be difficult because a safe method to address PV congestion by acute PV occlusion had not been established. Therefore, we moved forward to develop an antithrombogenic bypass catheter using heparinized hydrophilic polymer.^1^, ^2^, ^5^ The antithrombogenicity of this catheter is excellent. The whole inner surface of the catheter is coated with heparinized hydrophilic polymer, and the outer surfaces of both ends of the catheter that are inserted into the vessels are also coated with the same polymer.^2^, ^5^, ^7^ In case of PV occlusion, congested portal blood is effectively bypassed from one branch of the SMV into the systemic venous circulation, such as the femoral vein (Figure 1A, Figure 2) or inferior vena cava (Figure 1D) to prevent portal congestion and facilitate the PV resection and subsequent reconstruction without the time limitation of PV occlusion.^6^ When we attempted to perform the PV and hepatic artery resection and reconstruction concurrently, the congested portal blood was bypassed from one branch of the SMV into the recanalized umbilical vein in the hepatic round ligament (Figure 1B) or hepatic hilar PV (Figure 1C), avoiding both portal congestion and hepatic ischemia.^6^ Therefore, this procedure can dissolve the time limitation of PV occlusion. This catheter is now commercially available, and this procedure has become widespread throughout Japan.

## 3 ARTERIAL ANATOMY OF PERIPANCREATIC HEAD REGION

**FIGURE 3:**
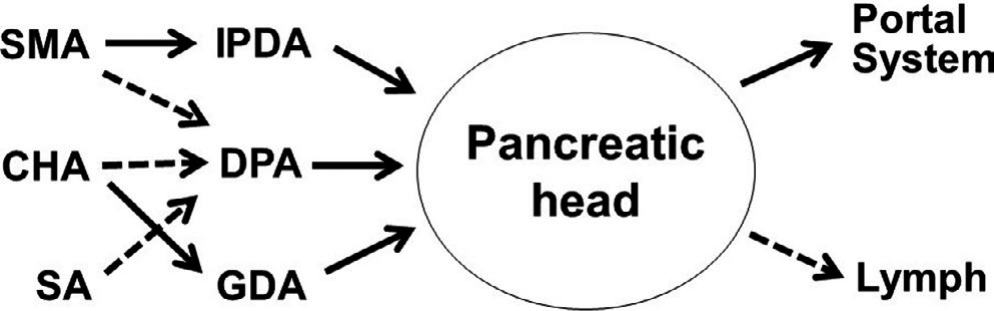
Blood supply of the pancreatic head region. SMA, superior mesenteric artery; CHA, common hepatic artery; SA, splenic artery; IPDA, inferior pancreaticoduodenal artery; DPA, dorsal pancreatic artery; GDA, gastroduodenal artery

Completion of isolated PD is technically difficult because of the complex arterial anatomy of the peripancreatic head region (Figure 3). In conventional PD using Kocher's maneuver, the gastroduodenal artery (GDA) can usually be ligated and divided during surgery; however, IPDA ligation and division is conducted in the final stage of the resection. In our isolated PD using the mesenteric approach, all branches from the SMA, including the MCA, JA2, JA1, IPDA, DPA, and rRHA, are gradually exposed, and both the IPDA and DPA are ligated and divided in the early stage of the PD. After performing the mesenteric approach, the GDA from the common hepatic artery (CHA) and the DPA from the CHA, celiac artery, or splenic artery can be ligated and divided in the usual manner.

## 4 SURGICAL TECHNIQUES IN ISOLATED PD USING MESENTERIC APPROACH

### 4.1 Supramesocolic mesenteric approach

The supramesocolic approach is a mesenteric approach that is usually indicated for cancer of the distal bile duct or duodenal papilla of Vater. After laparotomy by an upper midline skin incision, the gastrocolic ligament is divided and the lesser peritoneal cavity is fully opened. After the middle colic vein (MCV) and MCA become visible on the anterior surface of the transverse mesocolon, both the SMV and SMA are exposed along the roots of the MCV and MCA. Both the SMV and SMA are retracted with tape, and the connective tissues, including lymph nodes along the SMA, are dissected. By performing this procedure, the JA1 and IPDA are exposed along the right surface of the SMA. Preoperative multidetector computed tomography is essential to detect the location of the IPDA as well as other branches from the SMA, such as the MCA, DPA, rRHA, and jejunal arteries. In general, however, total excision of PLphII is unnecessary for cancer of the distal bile duct or papilla of Vater. As described above, the supramesocolic approach as a mesenteric approach makes it easy to accomplish systematic lymph node dissection around the SMA and early ligation of the IPDA or DPA from the SMA.

### 4.2 Inframesocolic mesenteric approach

The inframesocolic mesenteric approach is our typical approach for pancreatic head cancer.

#### 4.2.1 Laparotomy

Laparotomy is performed with use of an upper midline skin incision. The abdominal cavity is examined using washing cytology and intraoperative ultrasound. In general, the indication for surgical resection of pancreatic cancer is the absence of distant metastasis with a guarantee of cancer‐free surgical margins.

#### 4.2.2 Mesenteric incision and connective tissue clearance around SMV and SMA

**FIGURE 4:**
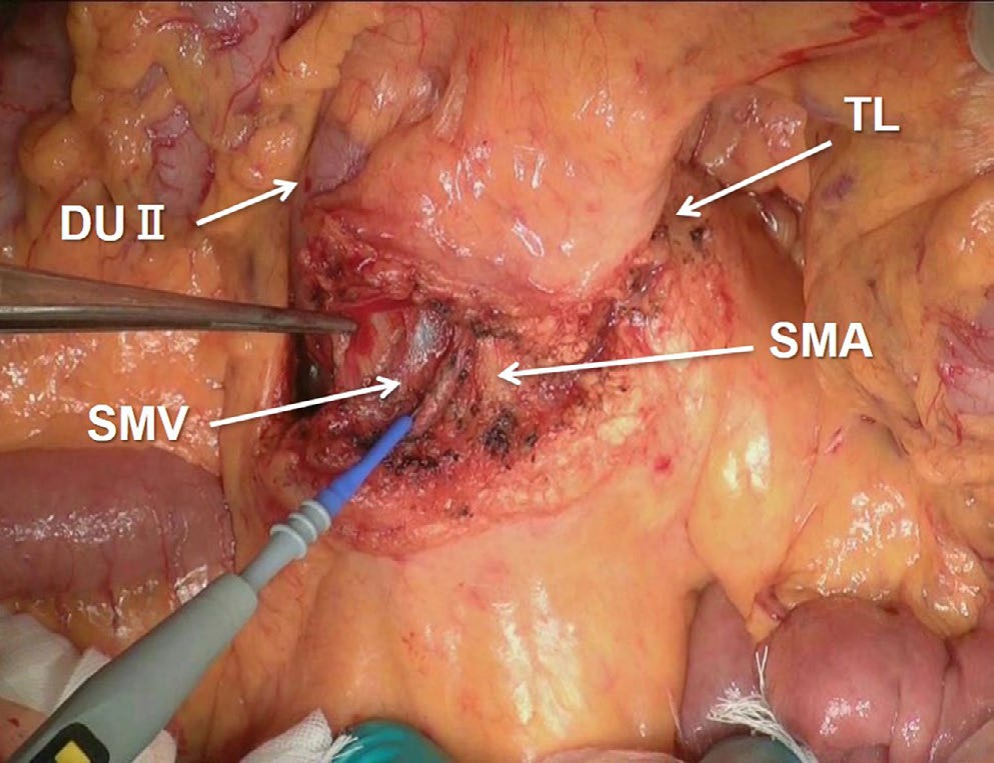
Mesenteric incision from the TL to the lower border of the DUII and connective tissue clearance around the SMV and SMA. TL, ligament of Treitz; DUII, second portion of the duodenum; SMV, superior mesenteric vein; SMA, superior mesenteric artery

**FIGURE 5:**
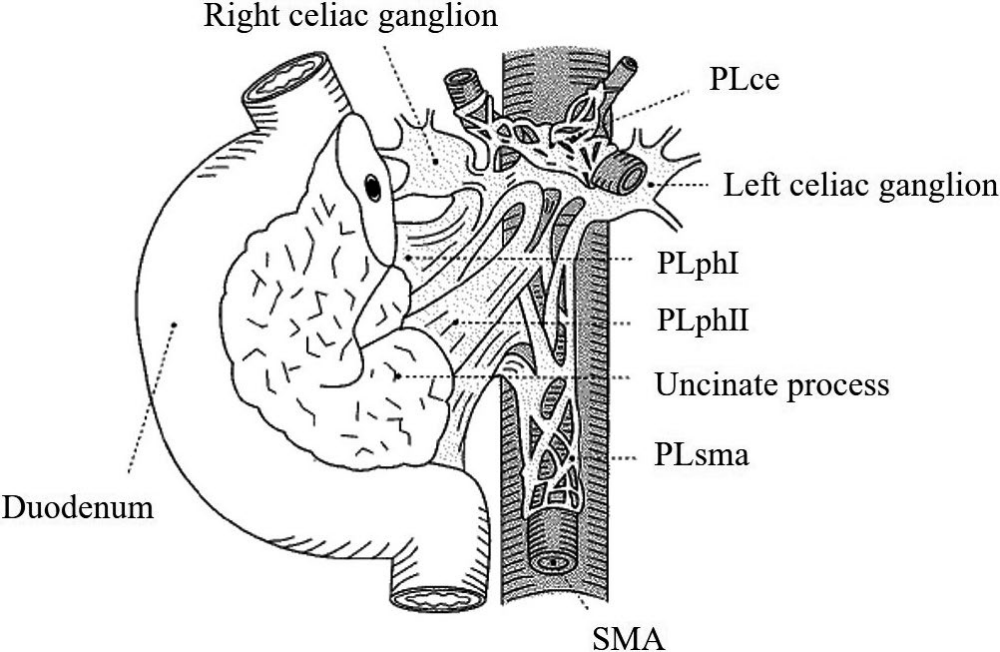
Extrapancreatic nerve plexus (from Reference^14^). PLce, celiac plexus; PLphI, first portion of the pancreatic head nerve plexus; PLphII, second portion of the pancreatic head nerve plexus; PLsma, nerve plexus around the superior mesenteric artery; SMA, superior mesenteric artery

The first step of our isolated PD is the mesenteric approach, and the first procedure of the mesenteric approach is incision of the mesentery from the ligament of Treitz to the lower border of the second portion of the duodenum. At this time, the surface of the mesentery is incised until exposure of the anterior walls of the SMV and SMA. In this first approach, Kocher's maneuver is never performed. All of the connective tissues, including the lymph nodes around the SMV and SMA (No. 14 lymph nodes),^14^, ^26^ are totally dissected to the lower border of the pancreatic head (Figure 4). Next, the MCA and MCV are gradually exposed on the anterior surface of the SMA and SMV. These branches are generally ligated and divided at the root; however, the marginal arcades of the MCA and MCV are carefully preserved. This approach makes it easier to conduct the connective tissue clearance around the root of the SMA than when the MCA and MCV are preserved. Of course, knowledge of the anatomy of the extrapancreatic nerve plexus (Figure 5)^14^, ^15^ is very important to achieve R0 resection.

#### 4.2.3 Exposure of PLphII, jejunal artery, IPDA, and DPA

**FIGURE 6:**
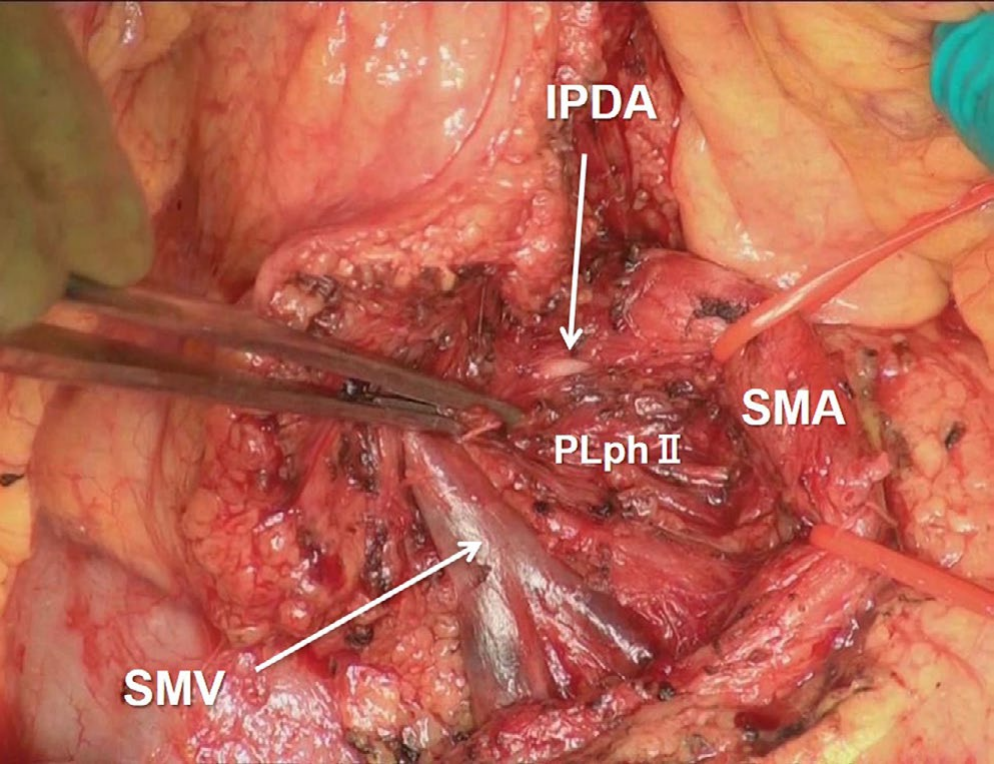
Exposure of the PLphII and IPDA. PLphII, second portion of the pancreatic head nerve plexus; IPDA, inferior pancreaticoduodenal artery; SMV, superior mesenteric vein; SMA, superior mesenteric artery

The connective tissue clearance around the SMV and SMA proceeds to the root of the SMV and SMA. At this time, the PLphII is clearly exposed between the SMA and SMV by appropriate traction of the SMA toward the left side and the SMV toward the right side (Figure 6). If no cancer invasion to the PLphII is observed, the nerve plexus around the SMA (PLsma) should be completely preserved to avoid postoperative diarrhea. If cancer invasion into the PLphII or PLsma is strongly doubted, the PLsma should be resected together with the PLphII to obtain a cancer‐free surgical margin. If it is thought to be difficult or impossible to obtain cancer‐free surgical margins, the radical resection should be stopped at this time. The radical resection should also be stopped when reconstruction of the SMV is considered to be impossible because of severe cancer invasion into the peripheral branches of the SMV.

The JA2 and JA1 generally lie behind the region of the SMA. The IPDA is generally a branch from the JA1 and lies within the region of the PLphII (Figure 6). However, many variations of the IPDA anatomy are well‐known, and the IPDA ligation and division in the early stage of PD may decrease the intraoperative blood loss.^27^ The DPA is sometimes observed as a branch from the SMA, and its ligation and division in the mesenteric approach may also decrease the intraoperative blood loss,^28^, ^29^ as can IPDA ligation and division. The rRHA can also be exposed by the mesenteric approach. In patients with more locally advanced pancreatic cancer, division of the pancreas along the line of the SMA or opening the transverse mesocolon might help to visualize the root of the SMA.

#### 4.2.4 Total excision of PLphII

**FIGURE 7:**
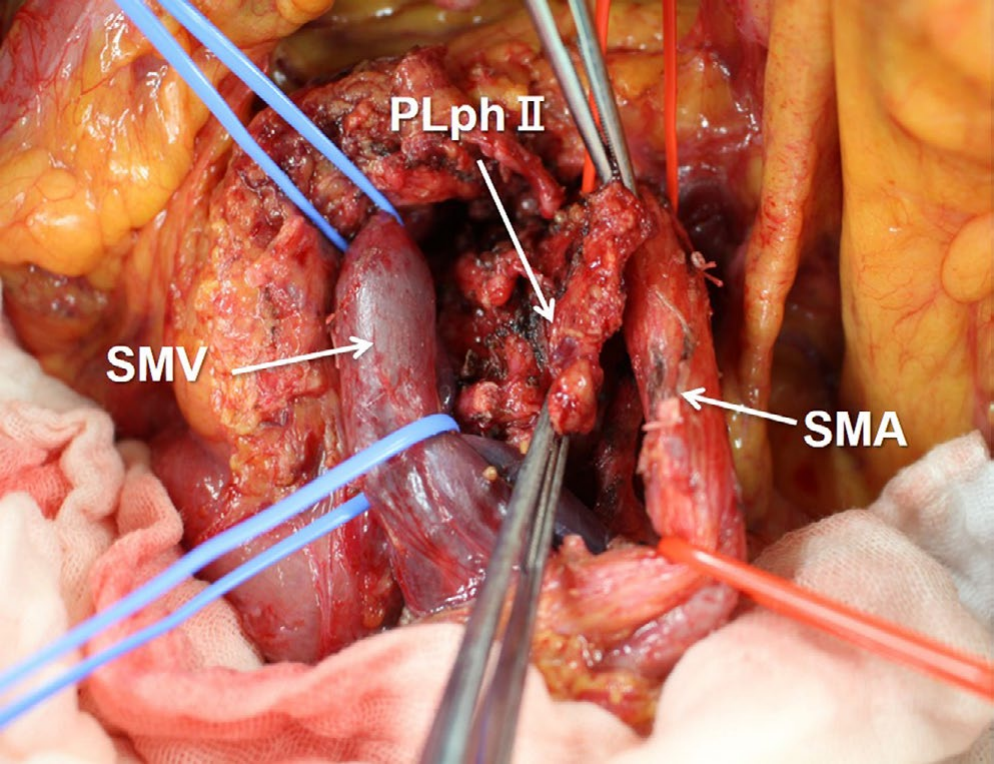
Total excision of the PLphII. PLphII, second portion of the pancreatic head nerve plexus; SMV, superior mesenteric vein; SMA, superior mesenteric artery

The connective tissue clearance around the SMV and SMA proceeds to the root of the SMV and SMA. The term “mesopancreas” has been in use since 2007 and was introduced by Gockel et al^13^; however, there is no precise anatomical definition of the mesopancreas. Actually, the anatomy of the extrapancreatic nerve plexus is precisely described in the Japanese classification of pancreatic cancer^14^ (Figure 5); therefore, we strongly propose that “mesopancreas” refers to the PLphII. Total excision of the PLphII from its attachment to the SMA (Figure 7) completes the mesenteric approach.

#### 4.2.5 Division of gastrocolic ligament and incision of transverse mesocolon

**FIGURE 8:**
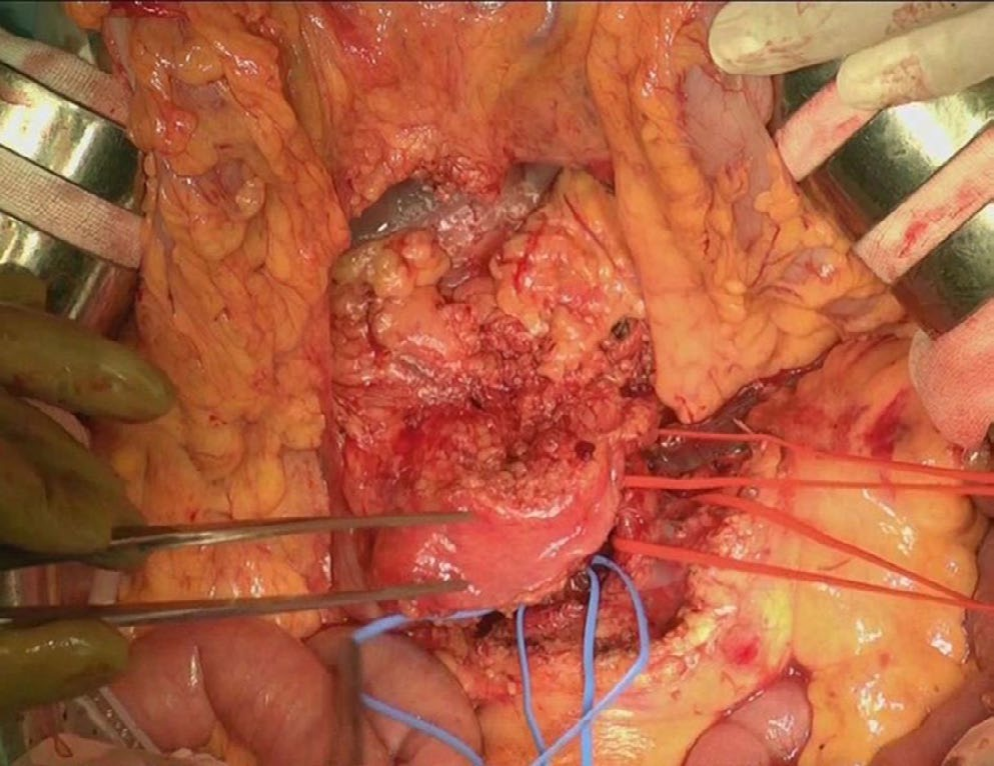
Incision of the root of the transverse mesocolon and en bloc dissection of the root of the mesenterium and mesocolon

The gastrocolic ligament is usually incised near the transverse colon, and the lesser abdominal cavity is fully opened. The transverse mesocolon can therefore be examined from both the anterior and posterior sides, and the anterior surface of the pancreas can be visualized. The root of the transverse mesocolon is horizontally incised and resected with preservation of the marginal arcade of the MCA and MCV. The connective tissue of the root of the mesentery and mesocolon is resected en bloc (Figure 8).

#### 4.2.6 Typical procedure after mesenteric approach to perform isolated PD

**FIGURE 9:**
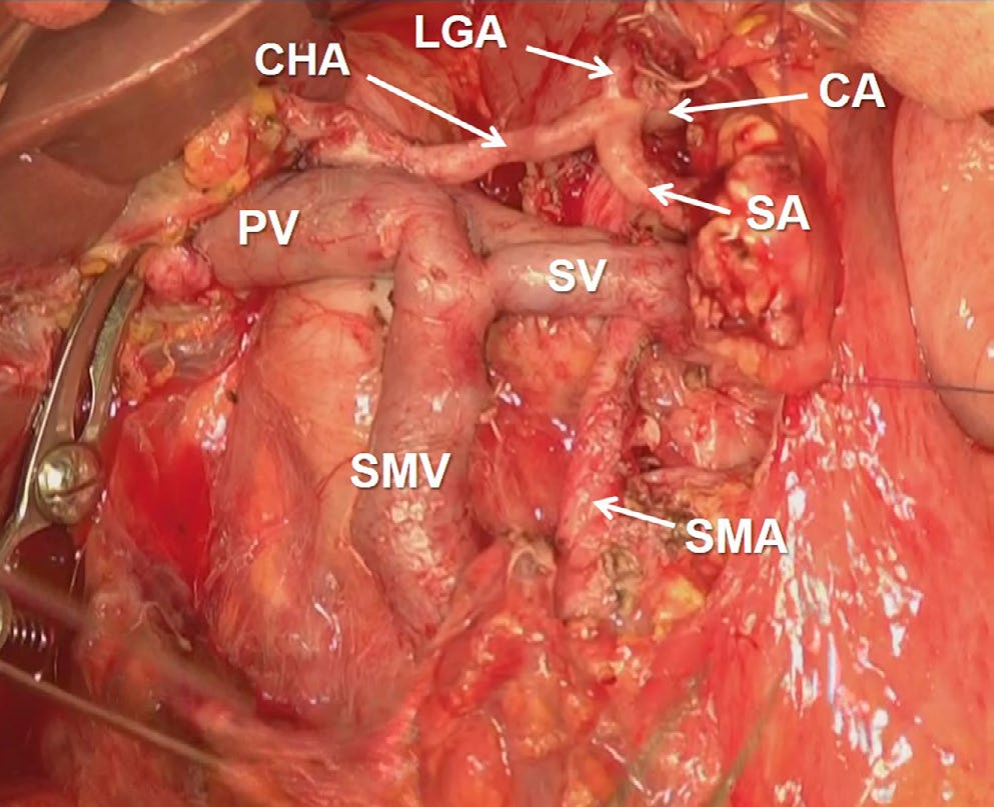
Conversion surgery using the mesenteric approach for unresectable locally advanced pancreatic head cancer after induction chemotherapy. CA, celiac artery; LGA, left gastric artery; CHA, common hepatic artery; SA, splenic artery; SV, splenic vein; PV, portal vein; SMV, superior mesenteric vein; SMA, superior mesenteric artery

After the completion of this mesenteric approach, the operative field is moved to the hepatic hilum area. The gallbladder is resected first. Complete clearance of the hepatoduodenal ligament including the lymph nodes (No. 12a, b, p)^14^ is performed, and the right gastric artery is ligated and divided. The GDA is then carefully ligated and divided. The stomach is usually divided at the prepylorus, and lymph node dissection around the CHA (No. 8a, p)^14^ and the celiac artery (No. 9)^14^ is conducted. Preoperative computed tomography angiography is extremely useful to differentiate the DPA from the CHA, celiac artery, or splenic artery. A complex peripancreatic arterial arcade is present in the pancreatic head region; therefore, controlling this whole arterial arcade is necessary to decrease the intraoperative blood loss during the isolated PD.^28^, ^29^ Next, the first portion of the pancreatic head nerve plexus (PLphI) is also dissected. At this time, if cancer invasion into the PV or SMV is observed, the PV or SMV can be resected and reconstructed. End‐to‐end anastomosis of the portal reconstruction can be easily performed by use of the mesenteric approach, avoiding tension. During the resection of the PV–SMV confluence, we advocate that splenic vein reconstruction is generally unnecessary and that preservation of left gastric vein is essential to reduce left‐sided portal hypertension.^30^, ^31^ When we use the antithrombogenic catheter‐bypass procedure of the PV, the catheter is extracted after the portal reconstruction. These procedures complete the isolated PD by the mesenteric approach (Figure 9).

## 5 DISCUSSION

Kocher's maneuver has historically been the first step in PD. We have developed this mesenteric approach based on our extensive experience with vascular resection using the antithrombogenic catheter‐bypass procedure for the PV in PD. In our opinion, isolated PD using this mesenteric approach and antithrombogenic catheter‐bypass procedure, if necessary, is the ideal surgery for the treatment of pancreatic head cancer from both surgical and oncological aspects. The mesenteric approach allows dissection from the non‐cancerous side and initial determination of cancer‐free margins and surgical resectability followed by systematic lymphadenectomy around the SMA.^26^ This approach can also enable early ligation of the IPDA and other branches from the SMA, such as the DPA, which can in turn reduce the venous congestion of the pancreatic head region and decrease the blood loss during surgery,^28^, ^29^ along with the ligation of the GDA and total excision of the PLphII and PLphI. Furthermore, this approach makes it easy and safe to resect and reconstruct the mesenteric vessels. These points are surgical merits of our mesenteric approach.

Why did we not use the term “artery‐first” in our description of this technique? The answer is that use of the artery‐first approach is common sense for surgeons who treat cancer. Therefore, we decided to use the term “isolated PD,” which refers to the performance of PD using a “non‐touch isolation” technique.^16^, ^17^ First, all arteries that flow into the pancreatic head region are ligated and divided; the veins from the pancreatic head region into the portal system are then ligated and divided; and finally, pancreatectomy is completely performed. We first published the description of this isolated PD using the mesenteric approach in 1993 in an English‐language journal^17^; however, this approach was not cited in other English publications.^32^, ^33^ It is possible that other journals did not notice our publication.^34^ The mesenteric approach enables systematic lymph node dissection and connective tissue clearance at the mesenteric root. Additionally, all branches from the SMA root, including the MCA, JA2, JA1, IPDA, DPA, and rRHA, are gradually exposed in the process of this approach. Thus, our mesenteric approach is absolutely different from the “artery‐first approach” in Western publications.

The oncological merits of the mesenteric approach might be the high incidence of cancer‐free margins, the systematic lymph node dissection around the SMA, no squeezing of cancer cells from the tumor by hand grasping, the low recurrence rate, and the high survival rate. However, no randomized controlled trials have compared the surgical and oncological merits of our mesenteric approach with the conventional Kocher's approach to PD. Nevertheless, in patients with resectable cancer of the pancreatic head, isolated PD using our mesenteric approach is suspected to result in a higher survival rate than that provided by conventional PD using Kocher's maneuver.^27^ Therefore, a randomized controlled trial is being performed in Japan to compare the surgical and oncological benefits of these two procedures.^35^

Because of recent advances in induction chemotherapy and chemoradiotherapy for patients with unresectable locally advanced pancreatic cancer, so‐called conversion surgery has become widespread. In this situation, the mesenteric approach and catheter‐bypass procedure of the PV are essential techniques for conversion surgery. The mesenteric approach has been gradually adapted throughout Japan, and by mastering the mesenteric approach and catheter‐bypass procedure of the PV, surgeons can successfully perform isolated PD.

## 6 CONCLUSION

We have herein described the precise surgical procedures of isolated PD using the mesenteric approach for treatment of pancreatic head cancer.

## CONFLICT OF INTEREST

None declared.
